# Exposure to needle stick injuries among health care workers in hemodialysis units in the southwest of Iran: a cross-sectional study

**DOI:** 10.1186/s12913-023-09465-w

**Published:** 2023-05-23

**Authors:** Jamshid Roozbeh, Leila Malekmakan, Mina Mashayekh, Anahita Dehghani, Soroush Ansari, Hossein Akbarialiabad, Mehdi Mahmudpour

**Affiliations:** 1 Department of Community Medicine, Shiraz Nephro-Urology Research Center, Shiraz University of Medical Sciences, Shiraz, Iran; 2 Global Health Academy, Nuvance Health, and the University of Vermont Larner College of Medicine, Vermont, United States; 3 St George and Sutherland Clinical School, UNSW Medicine, Sydney, Australia; 4 Persian Gulf Tropical Medicine Research Center, Persian Gulf Biomedical Sciences Research Institute, Bushehr University of Medical Sciences, Bushehr, Iran

**Keywords:** Needle stick injuries, Health care workers, Hemodialysis center

## Abstract

**Background:**

Needle stick injury (NSI) is the most common cause of infection with blood-borne pathogens (BBP) among healthcare workers (HCWs). This study aimed to assess the prevalence of NSI and it’s contributing factors among HCWs of hemodialysis (HD) units in southwest Iran.

**Methods:**

A cross-sectional study was performed in 13 HD centers in Shiraz, Iran. A total of 122 employees were enrolled in our study. We used self-administrated questionnaires to collect data about demographics, experiences regarding NSIs, and general health status. The statistical tests used in this study were Chi-square and Independent T-test. A *P*-value < 0.05 is considered significant.

**Results:**

The mean age of the study population was 36.1 ± 7.8 years (72.1%: women). Exposure to NSIs was reported by 23.0% of them at least once during the previous six months. NSI prevalence was significantly higher among those with higher age (*p* = 0.033), work experience > 10 years (*p* = 0.040), and those who graduated earlier (*p* = 0.031). The intravenous injection was the most common procedure leading to NSI, and being in a hurry was the most common cause. The average general health was 3.7 ± 3.2, higher among those not exposed to NSI (*p* = 0.042).

**Conclusion:**

NSI is a prevalent hazard in HCWs of HD units. The high rate of NSI and unreported cases, besides the lack of adequate information, indicates the necessity of implementing protocols and strategies for improving the safety of this personnel. It is difficult to compare the result of this study with those performed among HCWs in other settings; hence, further studies are needed to determine whether HCWs of these units are more exposed to NSIs.

## Introduction

Needle stick injuries (NSIs) refer to occupational exposure to a penetrating wound with another person’s body fluid [1]. According to the world health organization, 35 million healthcare workers (HCWs) encounter more than two million sharp injuries yearly [2]. Transmission of blood-borne pathogens (BBP), i.e., human immunodeficiency virus (HIV), hepatitis C virus (HCV), and hepatitis B virus (HBV), [3], is the major concern with 4.4%, 39.0%, and 36.7% global incidence, respectively associated with NSIs [1]. However, the psychiatric consequences of such injuries should also be considered [4]. Due to the high prevalence of BBP and the lack of proper equipment and policies in developing countries, HCWs in these regions are more endangered [5].

In addition, hemodialysis (HD) units have been considered highly endangered for biological risk [6]. Both patients and staffs in these units are at risk of blood-borne infections. In this setting, the type of invasive procedure nurses perform, such as inserting dialysis catheters or doing needle artery-venous fistulae by senior nurses, makes NSI a well-established occupational hazard among personnel. At the same time, technicians have issues with machine maintenance [5]. Also, using large-caliber puncture needles in HD units can transmit a large blood volume during an NSI event in this setting. Regarding BBP, some, like hepatitis C, are endemic among patients who underwent chronic dialysis. Another fact that can be attributed to NSIs, particularly in HD centers, is that due to the long incubation period of some blood-borne infections, patients can be a potential source of transmission; despite a negative test, therefore, regular testing of affected personnel is recommended for at least six months [7].

While many studies are performed among HCWs in different hospital wards, information about this hazard in personnel of dialysis centers, as a neglected population, is still restricted. To our knowledge, this cross-sectional study is the first survey in Iran to report the prevalence of NSIs among HCWs in all HD units in Shiraz metropolitan city. We aimed to identify the prevalence, predisposing factors, level of awareness, and whether HCWs of these units are more endangered. Also, preventive strategies and guidelines have been reviewed to improve decision-making policies and reduce this serious occupational hazard, especially in HD units.

## Methodology

### Study population

In this cross-sectional study, health personnel of all HD centers in Shiraz (13 centers, with 125 personnel) who worked during 2021 was enrolled. The inclusion criteria were at least 6-months of work experience as a nurse or dialysis technician in HD centers. Physicians of each stratum, such as residents and specialists, medical interns and students, and ancillary staff in HD centers, were not considered our target population. If the HCWs were unwilling to participate or were not present at the time of data collection, they were excluded from the study. After administering the questionnaire, our research team visited sample units on several occasions to assess the expected response rate. Finally, among 125 personnel who met the inclusion criteria, with a response rate of 97.6%, 122 completed questionnaires were returned.

### Instruments

***A structured self-administrated questionnaire-*** The required data were collected through a structured self-administrated questionnaire designed by the researcher (a self-made questionnaire) on demographic characteristics (age, sex, job category, level of education, health safety training, shift work, and use of protective equipment). The causes of NSI and other NSI-related factors were also extracted from a separate questionnaire. Notably, for some NSI-related parameters, such as the transmission of BBP, we also used self-statements of HCWs besides the questionnaire. The questionnaire was evaluated for validity and reliability. Content and face validity was confirmed by using a panel of experts (7 people). Reliability as external consistency was checked by Cronbach’s alpha, which was 0.812.

***General Health Questionnaire (GHQ-12)-*** The GHQ measures current mental health, and since its development by Goldberg in the 1970s, it has been extensively used in the Persian version. The scale asks whether the respondent has recently experienced a particular symptom or behavior. Each item is rated on a four-point scale (less than usual, no more than usual, rather more than usual, or much more than usual) [8, 9]. We obtained information about general health through a separate questionnaire(GHQ-12).

### Statistical analysis

Statistical data analysis was performed using Statistical Package for Social Sciences version 23 (SPSS Inc., Chicago, IL, USA). The results related to the continuous variables were presented as mean ± SD, and those related to the quantitative or categorical data were shown as numbers and percentages. The statistical tests used in this study were Chi-square and Independent T-test. A *P*-value < 0.05 is considered significant.

## Results

### Demographics

One hundred and twenty-two personnel, including 106 nurses and 16 dialysis technicians, completed the survey questionnaires, of which 72.1% (n = 88) were women. The age range of participants was between 23 and 59 years (Mean: 36.1 ± 7.8 years). The demographic characteristics of the study population are shown in (Table 1).

**Table 1:** Demographic characteristics of HCWs exposed to needle stick injuries

Characteristics	Total N = 122	Groups With NS Without NS		p-value
**Sex**				
Women Men	88(72.1) 34(27.9)	21(23.9) 7(20.6)	67(76.1) 27(79.4)	0.813
**Age**	36.1 ± 7.8	37.2 ± 8.2	35.7 ± 7.7	0.033
**Marital status**				
Married Single	97(79.5) 25(20.5)	22(22.7) 6(24.0)	75(77.3) 19(76.0)	1.000
**Education**				
AD^*^ Bachelor Master	16(13.1) 96(78.7) 10(8.2)	5(31.3) 20(20.8) 3(47.9)	11(68.8) 76(79.2) 7(70.0)	0.432
**Graduation**				
>20 years 10–20 years <10 years	37(30.4) 53(43.4) 32(26.2)	14(37.8) 9(17.0) 5(15.6)	23(62.2) 44(83.0) 27(84.4)	0.031
**Refractive disorders of eye**	50(41.0)	8(16.0)	42(84.0)	0.189
**Work experience**				
<5 years 5–10 years >10 years	30(24.6) 27(22.1) 65(53.3)	7(23.3) 3(11.1) 18(27.7)	23(76.7) 22(88.8) 47(72.3)	0.040
**Shift-work**				
>10 h <10 h Missing	89(73.0) 19(15.5) 14(11.5)	22(24.7) 4(21.1) 2(14.3)	67(75.3) 15(78.9) 12(85.7)	1.000
**Job category**				
Nurse Technician	106(86.9) 16(13.1)	21(19.8) 4(25.0)	85(80.2) 12(75.0)	0.162
**Hours of working/week**	49.1 ± 0.8	48.6 ± 0.5	50.2 ± 0.6	0.321
**Number of night shifts/month**	2.1 ± 0.4	2.0 ± 0.3	2.2 ± 0.5	1.000

AD, associate degree; HCW, health care worker; NS, needle stick

The statistical tests used in this study were Chi-square and Independent T-test

### Experiences regarding NSIs

About half of the studied population reported BBP (49 cases, 42.0%), most of which were through skin contact (41 patients, 33.6%), as HCWs reported. The prevalence of NSIs in our study population was 23.0% (n = 28), and 64.4% (18 cases) were exposed to NSIs at least once within six months before the study. Among exposed participants, 46.4% (13 cases) were exposed by others (during procedures in which more than one person was involved**)**, and in 82.1% (23 personnel), the dominant hand was injured. In addition, a higher exposure rate occurred in the morning shifts from 7 am-2 pm (42.8%, 12 persons).

When we considered the age, participants who experienced NSIs, were significantly older (37.2 ± 8.2 vs. 35.7 ± 7.7, *p* = 0.033). NSI was reported to be slightly higher among women, although it was insignificant (23.9% vs. 20.6%, *p* = 0.813). Those with higher work experience (> 10 years) were injured more frequently (27.7%, *p* = 0.040). Most injuries occurred in older-graduated personnel (37.8%, *p* = 0.031). No significant association was obtained between the occurrence of NSIs with the level of education (*p* = 0.432) or the hours of shift work (*p* = 1.000).

### The causes of NSI

In our study, the most common procedure leading to NSI was intravenous injection procedures (50.0%, 14 cases), followed by fistula manipulation (32.2%, 9 participants). In addition, being in a hurry (60.7%, 17 cases) caused the highest rate of injuries, followed by carelessness (21.4%, 6 patients). Regarding post-exposure actions, 82.1% (23 cases) of the respondents first washed the injury site with soap and water, and only 21.4% (6 cases) reported the event to the in-charge or center of infection control to get further evaluation or care. Of our study population, 79.5% were unaware of needle stick and sharp injury safety policies. Among participants, 70.5% checked and knew the HBV antibody serum titer. In this survey, 20 out of 28 samples studied who experienced NSI (71.4%) were less afraid of BBV (blood-borne virus), while 8 of them (28.6%) were so scared of getting infected (*p* = 0.010) (Table 2).

**Table 2:** Needle stick injuries by circumstance

Characteristics	Total (n = 28)
**NS**	
1 time 2 times 3 times	18(64.4) 5(17.8) 5(17.8)
**Needling procedure**	
Intravenous injection Sampling Fistula manipulation	14(50.0) 5(17.8) 9(32.2)
**Cause of exposure**	
Hurry Carelessness Inappropriate environment No reason	17(60.7) 6(21.4) 2(7.2) 3(10.7)
**Exposure by others (during team procedures)**	13(46.4)
**Injury of the dominant hand**	23(82.1)
**Exposure time**	
7am-14pm 14pm-20pm 20pm-7am	12(42.8) 8(28.6) 8(28.6)
**The first action**	
Wash hands with sanitizer Wash hands with soap Nothing	3(10.8) 23(82.1) 2(7.1)
**Inform infection control after injury**	26(92.9)

NS, needle stick

For prevention strategies, 12.3% of participants always used three types of protection, including gloves, gowns, and glasses; among them, only one person had experienced NSI (*p* = 0.022). Of our population, only nearly half (43.4%) reported that they had received enough safety training about NSIs (Table 3).

**Table 3:** Healthcare worker’s knowledge of needle stick injuries

Attitude	Completely agree	Slightly agree	No idea	Slightly disagree	Completely disagree
**Using glasses is useful**	97(79.5)	15(12.3)	6(4.9)	1(0.8)	0
**Using gloves is useful**	46(37.7)	30(24.6)	13(10.7)	12(9.8)	18(14.8)
**Having knowledge is useful**	96(78.7)	16(13.1)	4(3.3)	1(0.8)	1(0.8)
**Attends classes is useful**	81(66.4)	26(21.3)	11(9)	2(1.6)	0
**Using simulations is useful**	54(44.3)	40(32.8)	21(17.2)	5(4.1)	0
**Quantity and quality of training are enough**	26(21.3)	38(31.1)	17(13.9)	25(20.5)	11(9)
**I have received enough training**	53(43.4)	43(35.2)	10(8.2)	8(6.6)	4(3.3)

***General health-*** We also assessed the general health of our studied sample, with a mean average of 3.7 ± 3.2. Those who did not experience NSIs, were at a higher level of general health (4.0 ± 3.4 vs.3.5 ± 2.7 respectively, *p* = 0.042) (Table 4).

**Table 4:** The general health of healthcare workers according to the General Health Questionnaire (GHQ-12)

General Health	4	3	2	1	missing	C-GHQ 0 1
**Are you focused?**	23(18.9)	66(54.1)	16(13.1)	8(6.6)	9(7.4)	89(73.0) 24(19.7)
**Do you have insomnia?**	30(24.6)	30(24.6)	41(33.6)	14(11.5)	7(5.7)	60(49.2) 55(45.1)
**Do you feel useful?**	24(19.7)	70(57.4)	20(16.4)	0(0.0)	8(6.6)	94(77.0) 20(16.4)
**Are you able to make decisions?**	19(15.6)	79(64.8)	14(11.5)	1(0.8)	9(7.4)	98(80.3) 15(12.3)
**Are you under pressure?**	13(10.7)	36(29.5)	51(41.8)	12(9.8)	10(8.2)	49(49.2) 63(51.6)
**Do you feel can’t overcome the problems?**	31(25.4)	41(34.4)	35(28.7)	6(4.9)	8(6.6)	73(59.8) 41(33.8)
**Do you enjoy life?**	6(4.9)	60(49.2)	30(24.6)	18(14.8)	8(6.6)	66(54.1) 48(39.3)
**Can you face your problems?**	14(11.5)	71(58.2)	21(17.2)	7(5.7)	9(7.4)	85(69.7) 28(23.0)
**Do you feel depressed?**	32(26.2)	34(27.9)	34(27.9)	13(10.7)	9(7.4)	66(54.1) 47(38.5)
**Have you lost your confidence?**	52(42.6)	34(27.9)	23(18.9)	4(3.3)	9(7.4)	86(70.5) 27(22.1)
**Do you feel worthless?**	72(59.0)	24(19.7)	15(12.3)	2(1.6)	9(7.4)	96(78.7) 17(13.9)
**Are you happy?**	11(9.0)	57(46.7)	33(27.0)	11(9.0)	10(8.2)	68(55.7) 44(36.1)
**Total number**	**3.7 ± 3.2**					

## Discussion

Due to the incremental prevalence of hypertension (HTN) and diabetes mellitus (DM), population aging, and availability of dialysis, a rapid increase in the number of patients receiving dialysis has occurred worldwide; of them, 89% undergo HD [10]. We hypothesized that workforce staffing and potential risks for BBP transmission in HD centers, besides the rising trend in the number of patients undergoing HD, have made personnel of these units more exposed to occupational hazards such as NSIs. MacCleary et al. reported that HCWs in dialysis centers are twice as likely to be exposed to NSIs compared to other settings [11]. Procedures performed for dialysis access frequently, transfusion, and contamination in this setting are the main sources for transmission of infection [12]. Notably, according to a study performed in HD units in Cameroon, most participants (82.6%) had burnout syndrome, and 62% had decreased professional achievements [13].

Additionally, considering the large amount of transferable blood in each NSI [7] and the types of prevalent infective pathogens [3] in HD units, we think that in case of NSI occurrence, this issue is more concerning among HCWs of these units. Dialysis patients are more likely to have hepatitis C than the general population (8.4% vs. 1.8%), which is still a significant concern in patients with chronic kidney disease (CKD). The frequent need for invasive medical procedures in HD units and nosocomial transmission have exposed this population to a higher prevalence of HCV, with a global prevalence of 40% in developing countries [14]. In addition, during 1985–2000, the incidence of HIV developed ten times among this population [11].

To our knowledge, there is limited data about the epidemiology of NSIs among personnel of HD centers in Iran and other countries. And based on our search, only a few studies have been published, so we conducted this study as the first survey in Iran among this group in Shiraz.

We reported that 23% of our study population had experienced NSIs at least once during the past six months before the survey. While we reported a higher rate of NSI by increasing age, in a study by Alamgir et al., aging in HCWs has caused fewer cases of NSI [15]. Our analysis also revealed that higher work experience is significantly associated with more injuries. In line with our result, Kwanzaa et al. indicated that among new registered nurses in Trinidad, those with more years of experience were most exposed to NSI [16]. This may be due to higher years of graduation in personnel with higher work experience and a lack of informative courses to inform them about recent scientific guidelines about NSIs in HD units. In addition, we showed that in our study population, older graduation time leads to more NSIs. This can be attributed to the fact that despite a considerable increase in research about occupational hazards such as NSIs in low and middle-income countries during the last decades, due to inadequate political commitment, resource limitation, poor data collection systems, and executive power to regulate protocols [17], those who graduated earlier have not received updated information or training on this issue.

In line with our study, a study on Malaysian HCWs revealed that the morning shift was the most common time of occurring injuries [18]. Elsewhere, based on nine years of data about NSIs in Turkey, investigators reported that most injuries occurred between 8 am to 4 pm [19]. It may be because at 7:00 am, the end of work time for the night shift nurses, sleepiness peaks, leading to higher hazards [20] since night sleep quality can affect daytime sleepiness [21]. Contrary to our study, Johns et al. reported that HCWs on night shifts are at higher risk of NSI than daytime workers [22].

***NSI in HD units***- Comparing our results with other studies performed in dialysis units, the rate of NSIs in a multi-center Nigerian survey conducted in 2011 was approximately similar to ours, with a prevalence of 24.5% during the 12 months before the survey [5]. In an older study conducted in Italy during 1993–1994, among HCWs in nine dialysis units during one year, 12.7% of sharp injuries were reported. In this study, skin contamination (51.42%) was more prevalent than sharp injuries. According to this survey, removing dialysis needles was the most frequent circumstance that led to percutaneous injury [23]. Kabbash et al. reported a higher rate (48.6%) of NSI among HCWs of HD units in Egypt in one year [24]. This study’s higher number of NSIs can be attributed to the extended study period.

***NSI in other units***- This rate was higher (44%) in another study on 371 Iranian HCWs Sabermoghaddam et al. in North Khorasan [25]. In a meta-analysis that enrolled 6480 nurses in Iran, the prevalence of NSIs was 44% in one year [26]. In another analysis, the prevalence was 42.5% among Iranian HCWs [27]. Also, some previous studies reported a lower one-year prevalence of NSI; 19.1% by Bekele et al. in southeast Ethiopia [28], 19% by Jacob et al. in the United Arab Emirates [29], and 17.5% by Reda et al. in eastern Ethiopia [30].

***Report***- Alsabaani et al. reported a lower rate of NSI (11.57%) and also unreported injuries (52.7%) in 786 HCWs of Saudi Arabia during 12 months [31]. Our study’s reported cases to an infection control unit were twofold larger than NSI-positive personnel in Rasht, Iran (10%). In this study, being too busy with work at the time of exposure, not taking the life-threatening risk of such events seriously, fear of losing job security, time spent in post-exposure follow-up, and not having enough knowledge about appropriate reporting procedures were considered as some reasons for unreported cases [32]. Also, in a study in central Greece, unreported injuries among HCWs were lower (69.6%). Moreover, this study demonstrated that not being injured severely by sharp objects may be the reason for non-reporting events [33].

***Awareness***- Of our study population, 79.5% were unaware of needle stick and sharp injury safety policies. According to a study conducted in Sudan to evaluate nurses’ awareness about HD access care, 98% believed hand hygiene was necessary for HD centers before manipulation. Still, only 70% were adherent to it [34]. In another study performed in Khartoum state, Sudan, among HCWs of nine dialysis centers, most of the nurses (89.5%) had less than “good” knowledge before the educational intervention [35].

***Programs***- To minimize the risk of BBV transmission among HCWs in HD units, those in clinical contact with patients should present demonstrative documents on immunization against HBV. If they get infected, to work clinically should be monitored and have occupational health clearance. Furthermore, patients who are infected with HBV should not go under dialysis by non-immunized staff. Washing hands after each contact with patients, wearing disposable gloves and gowns, protecting eyes by safety spectacle, and covering cuts or abrasions are some behavioral recommendations to improve staff safety. For equipment protection, more than two-thirds capacity of sharp containers shouldn’t be filled [36]. Informing HCWs through training courses, employing trained personnel, continuous evaluation of performance, and preparing NSIs protocol are recommended to improve the performance regarding blood precautions [24].

***Limitations-*** Our study has some limitations. Due to underreported cases of NSIs, the true prevalence may be underestimated. Also, among occupational hazards leading to BBP, we only considered NSIs and further studies about splash accidents leading to skin or mucus contamination are needed. Last, regarding infection control, we didn’t determine HBV immunization status by serology.

***Conclusions-*** Exposure to NSIs was reported by 23% of our study population at least once during the previous six months. The prevalence of NSI was significantly higher among those with higher age, work experience, and those who graduated earlier. NSI is a prevalent hazard in HCWs of HD units in Shiraz, Iran. The high rate of NSI and unreported cases, besides the lack of adequate information, indicates the necessity of implementing protocols and strategies for improving the safety of this personnel. It is difficult to compare the result of this study with those performed among HCWs in other settings due to the restricted number of studies among HCWs of HD units. And the considerable difference in the duration of the study; hence to determine whether HCWs of these units are more exposed to NSIs or not, further studies are needed.

## Data Availability

The datasets used and analyzed during the current study are available from the corresponding author upon reasonable request.

## Abbreviations

BBP: Blood-Borne Pathogen
BBV: Blood-Borne Virus
CKD: Chronic Kidney Disease
DM: Diabetes Mellitus
GHQ: General Health Questionnaire
HBV: Hepatitis B Virus
HCV: Hepatitis C Virus
HCW: Health Care Worker
HD: Hemodialysis
HIV: Human Immunodeficiency Virus
HTN: Hypertension
NSI: Needle Stick Injury
